# Actomyosin contractility drives apical polarization and membrane transport during tubulogenesis

**DOI:** 10.21203/rs.3.rs-7665826/v1

**Published:** 2026-03-25

**Authors:** Yuji Mizotani, Edwin M. Munro

**Affiliations:** 1 Department of Molecular Genetics and Cell Biology, University of Chicago; Chicago, IL 60637, USA.

## Abstract

Lumen formation during tubulogenesis requires the large-scale redistribution of cellular material, but the in vivo mechanisms remain unclear. Using the ascidian notochord as a tractable model, we show that actomyosin contractility orchestrates sequential, spatially distinct modes of cortical and cytoplasmic transport to promote lumen initiation and growth: lateral actomyosin centralizes apical determinants on cell contacts to position apical lumens; cyclic detachment and inward contraction of actomyosin bundles from a basal equatorial contractile ring drives internalization and transport of basal membranes toward the apical lumen surface to enable lumen growth. Basal membrane internalization/transport and lumen growth require both Ezrin/Radixin/Moesin (ERM) proteins and extracellular matrix (ECM). ERM promotes equatorial contractility and transmits inward contractile forces to basal membranes, while broad attachment to ECM resists inward forces to promote equatorial detachment. These findings reveal how cells integrate cortical and cytoplasmic modes of transport, driven by actomyosin contractility, to enable rapid lumen formation and likely other types of cellular remodeling.

## Introduction

Epithelial tubes transport fluid and gas in organs such as the kidney, lung, and vasculature, and defects in their formation can be catastrophic for human health. One widespread mode of tubulogenesis, known as cord hollowing, involves the *de novo* formation of a central lumen from an initially solid mass of cells (1–3). Lumen formation begins with the enrichment of apical determinants, such as partitioning-defective proteins (PARs), to discrete regions within cell-cell contacts. The PARs in turn serve as local cues to direct the delivery of additional factors, such as anti-adhesive proteins, ion transporters, and water channels, which collectively promote lumen initiation, expansion, and fusion into a single fluid-filled tube. Lumen formation thus offers a powerful opportunity to understand how the large-scale redistribution of molecules and membranes drives the rapid remodeling of cellular domains (4–8). In Madin–Darby Canine Kidney (MDCK) cells, where this redistribution has been best characterized, the anti-adhesive podocalyxin and the apical determinant Crumbs are initially enriched on the basal surface and must translocate apically to establish the lumen (9–11). However, in general, the dynamic mechanisms that underlie large-scale molecular redistribution and cellular remodeling during lumen formation remain poorly understood.

**Fig. 1.**
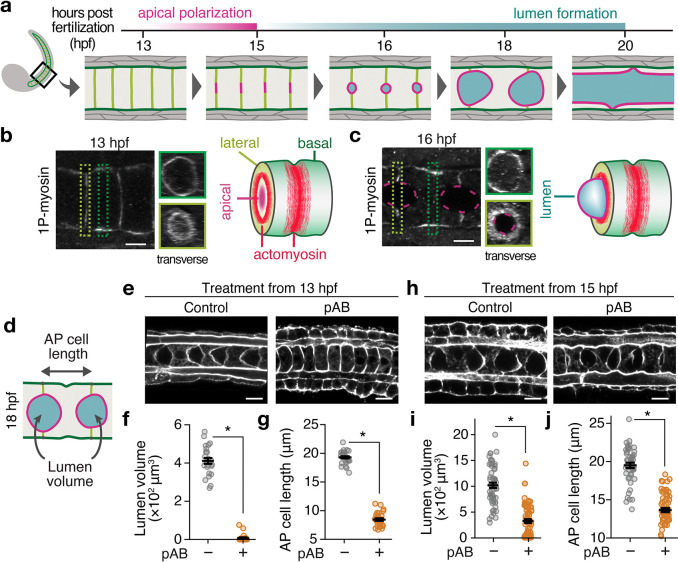
Myosin II activity is required for both lumen initiation and growth. **a** Schematic overview of ascidian notochord tubulogenesis. In this and all other panels, pink: apical domain; green: basal domain; olive: lateral domain; blue: lumen; **b**, **c** Immunostaining of 1P-myosin during (**b**) apical polarization (13 hpf) and (**c**) lumen formation (16 hpf) showing lateral (olive) and basal (green) pools of actomyosin. Dashed rectangles indicate axial positions used for transverse images. 3D schematics summarize the observed actomyosin activation patterns. **d**–**j** Effects of pAB treatment from 13 hpf (**e**–**g**) or 15 hpf (**h**–**j**) on cell elongation and lumen growth. (d) illustrates the measured quantities: anterior-posterior (AP) cell length and lumen volume. Representative micrographs (**e**, **h**) of control (DMSO-treated) and pAB-treated embryos stained with Alexa 488 Phalloidin to label F-actin. Plots below the micrographs show corresponding measurements of AP cell length (**f, i**) and lumen volume (**g, j**). Scale bars: 10 μm in (**b**); 5 μm in (**e**) and (**h**). Horizontal bars in (**f**), (**g**), (**i**), and (**j**) show mean ± SEM (Welch’s t-test). *P < 0.001.

Spatiotemporal control of actomyosin contractility drives deformation and transport during polarization and cellular remodeling in many different contexts (12, 13). During lumen formation, apical contractility is thought to resist lumen expansion (14–16), but whether and how actomyosin contractility acts outside the apical domain to drive large-scale transport and cellular remodeling remains unknown. Here, we use the ascidian notochord as a simple model system (17–19) to identify two modes of non-apical actomyosin contractility that drive key steps in lumen formation (Fig. 1a). Combining targeted perturbations with quantitative 3D microscopy, we show that lateral contractility concentrates PAR proteins on lateral contacts to establish new apical domains, while local assembly, detachment, and inward contraction of a basal contractile ring drives bulk basal-apical flow of membranes and other material to enable rapid lumen expansion.

## Results

### Actomyosin contractility is required for both lumen initiation and lumen growth.

From 13 to 20 hours post-fertilization (hpf), a sequence of steps transforms the notochord from a solid rod of coin-shaped cells into a fluid-filled tube (Fig. 1a). Apical polarization and deadhesion initiate small lumens on individual cell contacts (13–15 hpf). These individual lumens grow (15–18 hpf); then they meet and fuse (18–20 hpf) to form a single tube. Lumen initiation, growth, and fusion are accompanied by axial elongation and rearrangements of individual notochord cells (Fig. 1a). Here, we focus on lumen initiation and growth from 13–18 hpf.

Extending previous work (20–24), using phospho-specific antibodies against the active form of myosin II (1P-myosin), we detected basal and lateral activation of myosin II in notochord cells during both lumen initiation and lumen growth (Fig. 1b, c). Basal 1P-myosin is enriched at the equator within a cytokinetic ring-like structure that is thought to drive anterior-posterior (AP) cell elongation (21), while lateral myosin II is thought to restrict lateral expansion of lumen openings during early lumen growth (23, 24). To better distinguish stage-specific requirements for myosin II, we treated embryos with the myosin II inhibitor para-aminoblebbistatin (pAB), from either before (13 hpf) or after (15 hpf) apical polarization, and measured lumen volume and AP cell length at 18 hpf (Fig. 1d). Early inhibition blocked both lumen initiation and cell elongation (Fig. 1e–g), while late inhibition reduced lumen growth and impaired cell elongation (Fig. 1h–j). We observed similar effects upon early or late treatment with H1152, a Rho kinase (ROCK) inhibitor (Supplementary Fig. 1a–f). Thus, the ROCK-myosin pathway is required for two distinct processes—early to initiate lumen formation and later to drive its growth, concurrent with its effects on cell elongation.

### Actomyosin is required to polarize apical PARs on cell-cell contacts.

**Fig. 2.**
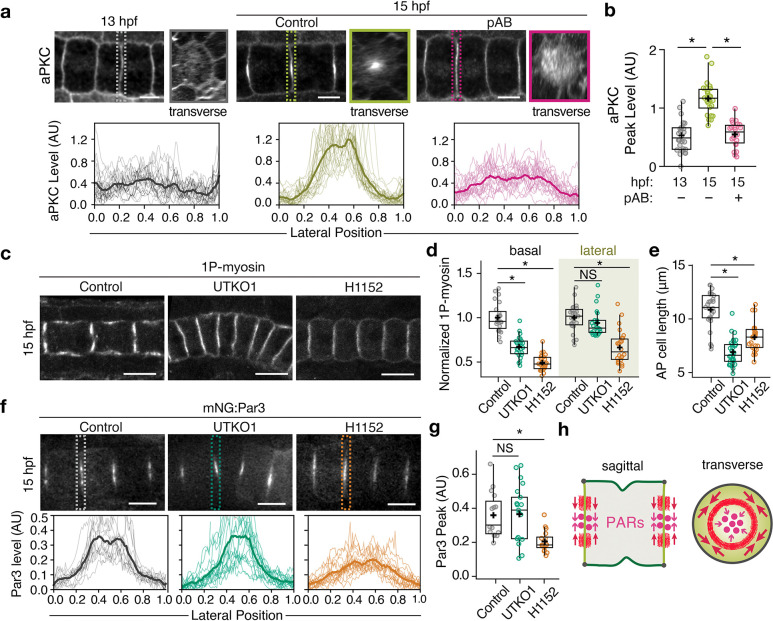
Lateral actomyosin is required for apical polarization. **a** (Top) Distributions of immunostained aPKC before (13 hpf) and after (15 hpf) the treatment with control (DMSO) or pAB; dotted rectangles indicate positions at which transverse sections were taken. (Bottom) Quantification of lateral aPKC signal intensities. Thin lines indicate individual measurements, and thick lines show the average. **b** Boxplots showing the aPKC peak levels. **c** 1P-myosin staining of embryos treated with control (DMSO), UTKO1 and H1152 from 13 hpf and fixed at 15 hpf. **d**, **e** Quantification of basal and lateral 1P-myosin levels (**d**) and AP cell elongation (**e**) for the same treatments. **f** (Top) mNG:Par3 at 15 hpf after treatment with control (DMSO), UTKO1, and H1152 from 13 hpf. (Bottom) Quantification of the mNG:Par3 lateral intensities for the same treatments. **g** Peak mNG:Par3 levels for the same treatments. **h** Schematic illustration showing how contraction of lateral actomyosin induces centralization of apical PARs. Scale bars: 5 μm in (**a**) and 10 μm in (**c**) and (**f**). In boxplots, horizontal lines indicate median, + indicate mean, boxes indicate second and third quartiles, and whiskers indicate 95% confidence intervals (Mann-Whitney U test with Bonferroni’s correction). NS, not significant; *P < 0.001.

We asked whether the failure to initiate lumens in pAB- or H1152-treated embryos was due to loss of apical polarization. In fixed immunostained control embryos, aPKC is uniformly localized on all notochord cell surfaces at 13 hpf and becomes highly enriched at the center of lateral contacts between cells by 15 hpf (Fig. 2a,b, (25)). By contrast, in embryos treated with pAB from 13 hpf onwards, aPKC remained broadly distributed on lateral contacts at 15 hpf. pAB treatment from 13–15 hpf also blocked centralization of fluorescent reporters for Par3 and Par6 (Supplementary Fig. 2a–c). Thus, actomyosin contractility is required to polarize apical PAR proteins on lateral contacts, a key prerequisite for lumen initiation.

### Identification of basal-selective myosin II inhibitors to isolate spatial functions of contractility.

**Fig. 3.**
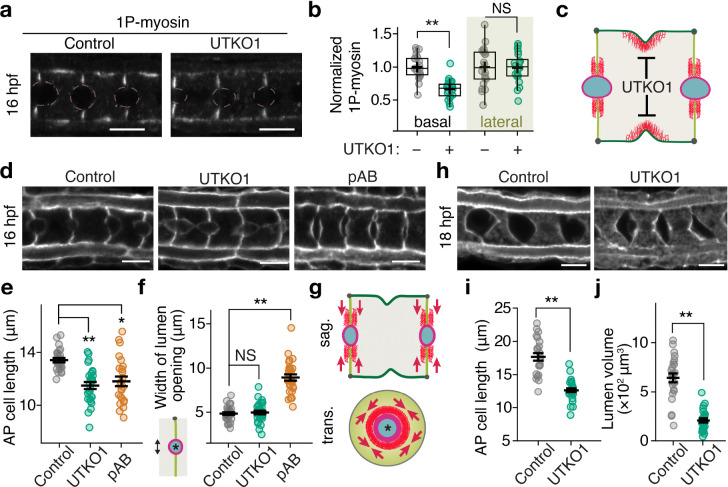
Basal actomyosin contractility promotes overall lumen growth. **a** 1P-myosin staining in embryos treated with control (DMSO) or UTKO1 from 15 hpf and fixed at 16 hpf. Pink dashed lines indicate the positions of lumens. **b** Quantification of basal and lateral 1P-myosin levels. **c** Schematic illustration showing the mode of action of UTKO1 on actomyosin contractility in notochord cells. **d** Representative micrographs of fixed phalloidin-stained embryos treated with control (DMSO), UTKO1, or pAB from 15–16 hpf. **e, f** Quantifications of AP cell length (**e**) and lumen opening (**f**) for the same treatments. **g** Schematic illustration showing the role of lateral actomyosin in resisting lumen opening. Red arrowheads indicate actomyosin contractility that opposes the pressure of lumen expansion. **h** Representative micrographs of fixed phalloidin-stained embryos treated with control (DMSO) or UTKO1 from 15–18 hpf. **i**, **j** Quantification of lumen volume (**i**) and cell length (**j**) for the same treatments. Scale bars: 10 μm. In (**b**), horizontal lines indicate median, + indicate mean, boxes indicate second and third quartiles, and whiskers indicate 95% confidence intervals (Mann-Whitney U test). In (**e**), (**f**), (**i**), and (**j**), horizontal bars show mean ± SEM (Welch’s t-test). NS, not significant;*P<0.01, **P < 0.001.

To delineate specific roles for basal and lateral actomyosin, we sought to identify selective inhibitors of basal contractility in ascidian notochords. We previously showed that the small molecule UTKO1 (26) inhibits ascidian notochord elongation and lumen formation (27), while the Polo-like kinase 1 (PLK1) inhibitor BI 2536 inhibits cytokinetic ring formation across species (28), identifying both as candidate inhibitors of basal myosin II. When applied during apical polarization (13–15 hpf) or lumen growth (15–16 hpf), both compounds reduced basal—but not lateral—myosin II activation (UTKO1: Fig. 2c, d, Fig. 3a–c; BI 2536: Supplementary Figs. 3b, 4a, b). In contrast, H1152 suppressed myosin II activation in both basal and lateral domains (Fig. 2c, d and Supplementary Fig. 4a, b). These results establish UTKO1 and BI 2536 as selective inhibitors of basal contractility; thus, comparing their effects with those of global inhibitors pAB and H1152 enables functional separation of roles for basal and lateral actomyosin.

### Lateral contractility concentrates apical PARs on cell contacts.

To distinguish contributions of basal and lateral actomyosin to polarization, we treated embryos with global or basal-selective inhibitors from 13–15 hpf. H1152 treatment blocked both cell elongation (Fig. 2e) and central accumulation of apical Par3 (Fig. 2f, g) by 15 hpf, as previously observed with pAB (Fig. 2a, b). In contrast, treatment with UTKO1 or BI 2536 inhibited cell elongation but did not affect PARs centralization (UTKO1: Fig. 2e–g; BI 2536: Supplementary Fig. 3c–f). These results confirm the previously proposed role for basal contractility in driving AP cell elongation (21) and identify a key role for lateral contractility in concentrating apical PARs at junction centers before lumen initiation (Fig. 2h).

### Basal contractility promotes lumen growth.

To distinguish roles for basal and lateral actomyosin during lumen growth, we treated embryos with global or basal-selective myosin II inhibitors and assessed early lateral opening (15–16 hpf) and lumen growth (15–18 hpf). Consistent with previous findings (23, 24), brief treatment with pAB or H1152 from 15–16 hpf enhanced early lateral lumen opening, whereas UTKO1 or BI 2536 did not, despite effectively inhibiting cell elongation (UTKO1: Fig. 3d–f; BI 2536: Supplementary Fig. 4c–e). However, prolonged treatment with UTKO1 or BI 2536 from 15–18 hpf impaired both lumen growth and cell elongation (UTKO1: Fig. 3h–j; BI 2536: Supplementary Fig. 4f–h), as observed with pAB (Fig. 1h–j) and H1152 (Supplementary Fig. 1d–f). These findings identify a novel and distinct role for basal contractility in promoting lumen expansion during 15–18 hpf.

### Basal actomyosin periodically contracts inward toward the cytoplasm and apical domain.

**Fig. 4.**
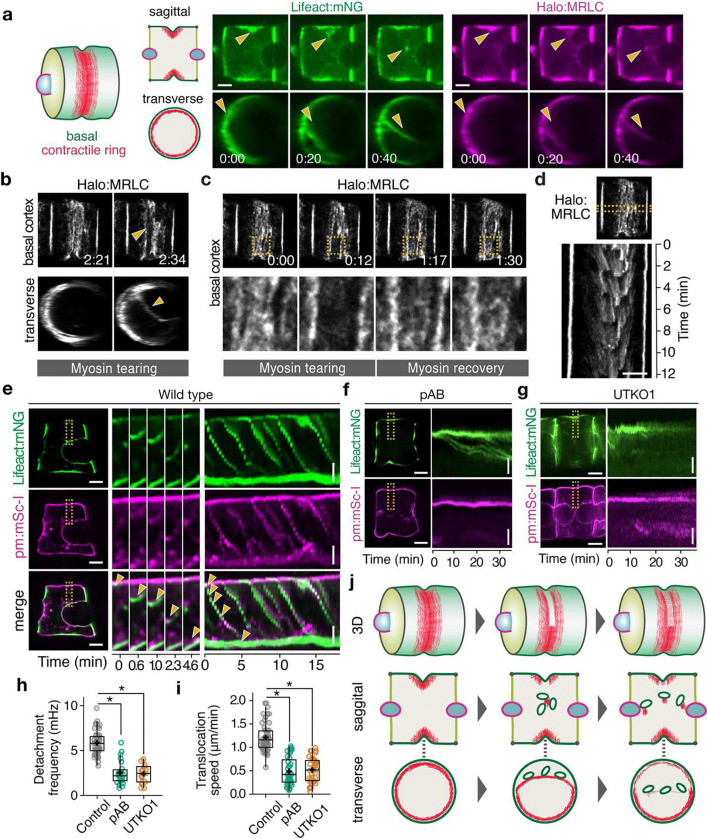
A basal contractility cycle drives internalization and basal-apical transport of basal material. **a** (Left) Schematic illustrations of notochord cells showing the basal contractile ring in 3D, sagittal and transverse views; (Right) corresponding images of notochord cells co-expressing Lifeact:mNeonGreen (mNG)(green) and Halo:MRLC (magenta). Yellow arrowheads indicate sections of the basal actomyosin ring undergoing internalization. **b** (top) Basal and (bottom) transverse views of the basal contractile ring notochord cells expressing Halo:MRLC. Yellow arrowheads indicate a section of the basal actomyosin ring undergoing internalization. **c** Sequential basal surface views of the same cell showing local tearing and recovery of the contractile ring. Bottom row shows magnified view of the region in the yellow boxes. **d** Kymograph for the region indicated by the horizontal rectangle illustrating repetitive cycles of tearing and self-repair in the cell shown in (**b**) and (**c**). **e** Basal-apical co-transport of F-actin and membranes in notochord cells co-expressing Lifeact:mNG (top row; green), and membrane marker (pm:mSc-I; middle row, magenta). Bottom row shows the two signals merged. In each row, left panel shows a single cell; middle panel shows expanded views of the indicated region (dashed yellow rectangle) over time illustrating one cycle of basal-apical transport; right panel shows a kymograph for the same region highlighting repetitive cycles of basal-apical transport. Yellow arrowheads in bottom row indicate the basal-apical movement of F-actin and membrane. **f**–**g** Sectional views and corresponding kymographs of embryos co-expressing Lifeact:mNG and pm:mSc-I and treated with pAB (**f**) and UTKO1 (**g**). Yellow rectangles indicate the region used for making kymographs. **h, i** Quantification of frequency (**h**) and speed (**i**) of basal-apical movements of actomyosin in embryos treated with control (DMSO), pAB and UTKO1. **j** Schematic summary of how the local detachment and inward contraction of basal actomyosin rings drive the internalization and apically directed movement of basal membranes. Scale bars: 5 μm in horizontal lines in (**a**), (**d**), (**e**) and (**f**); 2 μm in vertical lines in (**e**)–(**g**). In (**h**) and (**i**), horizontal lines indicate median, + indicate mean, boxes indicate second and third quartiles, and whiskers indicate 95% confidence intervals (Mann-Whitney U test with Bonferroni’s correction). NS, not significant; *P < 0.001.

These results raise a key question: how does basal contractility promote apical lumen expansion? Exploiting the semitransparency of ascidian embryos, we established multicolor 3D live imaging to monitor actomyosin dynamics using notochord-specific expression of fluorescently-tagged Lifeact and myosin regulatory light chain (MRLC) as reporters for F-actin and myosin II. We immobilized embryos at 16–17 hpf in agarose and imaged them with spinning disk confocal microscopy. Consistent with previous reports (20, 22), F-actin and myosin II were enriched in a basal contractile ring that is fed by a continuous flow of actomyosin from lateral contacts. However, 3D analysis revealed an unexpected behavior in which sections of the ring detach from the basal surface and move inward toward the apical surface of the expanding lumen (Fig. 4a, Supplementary Movie 1). Detached sections shorten and straighten as they move inward, indicating that these movements are driven by active contraction. Extended time-lapse imaging revealed repeated cycles of ring detachment and inward contraction (Supplementary Fig. 5a, Supplementary Movie 2). We observed similar dynamics when embryos were immobilized with anesthetic (MS-222; Supplementary Fig. 5b, Supplementary Movie 3), instead of agarose embedding, and using alternative probes for F-actin in live (utrophin; Supplementary Fig. 5c, Supplementary Movie 4), and fixed (phalloidin; Supplementary Fig. 5d) embryos. To further elucidate these dynamics, we used STED super-resolution imaging of MRLC. We observed two distinct forms of mechanical rupture: one in which sections of the contractile ring tear *away from* the basal cortex and contract inward (Fig. 4b, Supplementary Movie 5), and one in which local tears within the surface plane are followed by rapid lateral retraction of the contractile ring, which remains associated with the basal surface (Supplementary Fig. 5e). In both cases, myosin II was rapidly recruited to the site of the recent tear to repair the contractile ring (Fig. 4c), implying a local mechanism to sense and restore loss of mechanical continuity in the contractile ring, as reported in other systems (29). This cycle of local tearing and self-repair occurred episodically, with an average frequency of 0.35 min^−1^, from 16–18 hpf (Fig. 4d).

### Basal actomyosin drives large-scale membrane internalization and translocation.

Remarkably, co-imaging of F-actin and membranes revealed that the periodic detachment and inward contraction of sections of the contractile ring coincided with the internalization of correspondingly large patches of basal membrane and their translocation toward the apical domain (Fig. 4e, Supplementary Fig. 5f, Supplementary Movies 6, 7). Global inhibition of myosin II with pAB induced an anterior shift in basal ring position (Supplementary Fig. 6a, Supplementary Movie 8), consistent with previous reports (20, 22), and blocked both tearing and recovery of the ring, reduced the frequency and velocity of inward actomyosin movements, and blocked the internalization and translocation of basal membranes (Fig. 4f, h, i, Supplementary Movie 9). Similarly, UTKO1 and BI 2536 treatment impaired the formation of basal contractile rings and thus membrane internalization (UTKO1: Fig. 4g–i, Supplementary Movie 10; BI 2536: Supplementary Fig. 6b–d, Supplementary Movie 11). Notably, DIC microscopy previously revealed periodic flows of cytoplasmic elements from the basal equator toward the apical domain, which were abolished by UTKO1 (27)—highlighting the large-scale nature of actomyosin-driven translocation. Thus, repetitive assembly, local detachment and inward contraction of basal actomyosin drives the internalization and translocation of basal membranes toward the apical domain (Fig. 4j).

### ERM is essential for both myosin activation and membrane internalization.

**Fig. 5.**
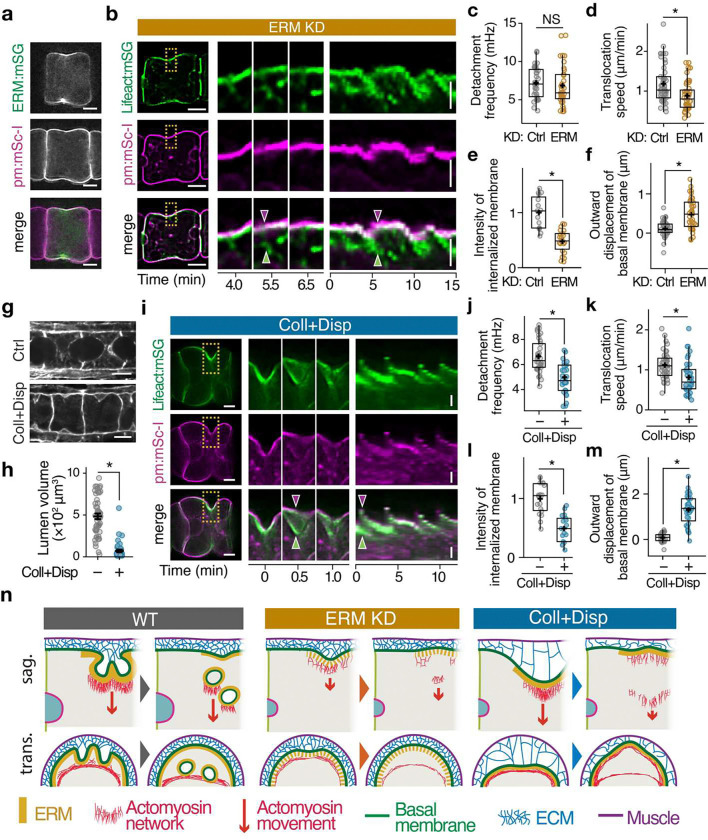
ERM proteins and extracellular matrix synergize to promote internalization and basal-apical transport of basal membranes. **a** Localization pattern of ERM:mSG (top), pm:mSc-I (middle), and the merged signals (bottom). **b** Cross-sections (left), magnified snapshots (middle) and kymographs (right) showing basal-apical movements of F-actin (Lifeact:mNG; top, green), membranes (pm:mSc-I, middle, magenta), and both (merged signals, bottom) in embryos injected with ERM morpholino (MO). Yellow rectangles at left indicate regions used for magnified snapshots and kymographs. Green arrowheads indicate detached and internalized sections of the basal contractile ring; magenta arrowheads indicate outward displacement of the basal membrane just after local detachment of the actin ring from the basal surface. **c, d** Quantification of frequency (**c**) and speed (**d**) of actomyosin movement in embryos microinjected with control or ERM MO. **e** Quantification of membrane signal colocalized with internalized F-actin. **f** Quantification of outward displacement of basal membrane after local detachment of basal F-actin from the surface. **g** Micrographs of the embryos at 18 hpf treated with or without Coll+Disp from 15 hpf. **h** Quantification of lumen volume in the embryos treated with or without Coll+Disp. **i** Micrographs and their kymographs of Lifeact:mNG (top, green), pm:mSc-I (middle, magenta), and their merged images of the embryos at 16–17 hpf treated with Coll+Disp from 15 hpf. Yellow rectangles at left indicate regions used for magnified snapshots and kymographs. Green arrowheads indicate detached and internalized sections of the basal contractile ring; magenta arrowheads indicate outward displacement of the basal membrane just after local detachment of the actin ring from the basal surface. **j**, **k** Quantification of frequency (**j**) and speed (**k**) of basal-apical movements of actomyosin in embryos treated with or without Coll+Disp. **l** Quantification of basal membrane internalization. Same as (e). **m** Quantification of outward displacement of basal membrane after local detachment of basal F-actin from the surface. **n** Schematic illustration of the dynamics of actomyosin and membrane in WT, ERM KD, or Coll+Disp treated embryos. Scale bars: 5 μm in horizontal lines in (**a**), (**b**), (**g**) and (**i**); 2 μm in vertical lines in (**b**) and (**i**). In (**c**), (**d**), (**e**), (**f**), (**j**), (**k**), (**l**), and (**m**), horizontal lines indicate median, + indicate mean, boxes indicate second and third quartiles, and whiskers indicate 95% confidence intervals (Mann-Whitney U test). In (**h**), horizontal bars show mean ± SEM (Welch’s t-test). NS, not significant; *P < 0.001.

Ezrin/Radixin/Moesin (ERM) proteins are broadly conserved actin–membrane linker proteins that can also modulate the ROCK–actomyosin pathway (30). Ascidians express a single isoform, ERM, which is enriched in the basal equatorial ring in notochord cells (Fig. 5a, (27)) and, similar to other systems (30–32), is required for lumen formation (33), suggesting that ERM could promote lumen growth by promoting basal contractility and/or by coupling basal contractility to membrane internalization. Suppressing ERM synthesis with morpholino (ERM-MO) reduced basal contractility and AP cell elongation, without affecting lateral contractility or apical PAR polarization (Supplementary Fig. 7a–c). Multicolor live imaging in ERM-MO embryos revealed that residual basal contractility is still associated with episodic local detachment and inward movement of equatorial F-actin (ERM-MO: Fig. 5b, Supplementary Movie 12; control-MO: Supplementary Fig. 7e, Supplementary Movie 13). However, while the frequency of detachment was similar to controls, the rate and extent of F-actin movements following detachment were sharply reduced (Fig. 5c, d), consistent with reduced levels of basal myosin activity (Supplementary Fig. 7a). More crucially, these F-actin movements were no longer coupled with membrane internalization (Fig. 5b,e, Supplementary Movie 12). Instead, local F-actin detachment was followed by locally outward displacement (blebbing) of basal membranes (Fig. 5b, f, Supplementary Fig. 7d, Supplementary Movie 14), as observed elsewhere (34), and indicating a loss of mechanical linkage between the equatorial cortex and the overlying basal membrane. These results suggest that ERM promotes apical lumen growth both by activating basal contractility and by mechanically coupling contractility to membrane internalization.

### ECM supports membrane internalization and transport driven by basal contractility.

In ascidian embryos, a collagen-rich extracellular matrix (ECM) accumulates between the basal notochord surface and surrounding muscle cells (35), and its inhibition reduces AP cell elongation (35), suggesting that basal ECM may modulate basal contractility and lumen growth. Consistent with this possibility, acute disruption of ECM, by treating embryos with collagenase and dispase (Coll+Disp) from 15 hpf, blocked lumen growth (Fig. 5g, h). While Coll+Disp treatment did not affect basal myosin II activity or contractile ring formation (Supplementary Fig. 8a, b), it had profound effects on the dynamics of ring detachment and basal membrane internalization and translocation. In Coll+Disp treated embryos, basal contractility induced broad, deep ingressions of the basal surface (Fig. 5i, Supplementary Fig. 8c, Supplementary Movie 15), consistent with loss of mechanical attachment to the overlying ECM (35). Ring detachment was delayed (Fig. 5j), and detachment events were followed by local blebbing of basal membranes (Fig. 5i, m, Supplementary Fig. 8d, e, Supplementary Movie 15–17), indicating complete loss of mechanical linkage between the detached ring and the overlying basal membrane. Consistent with this, the internalization of basal membranes was strongly attenuated (Fig. 5l). Inward contraction of detached actomyosin and translocation of internalized basal membranes were also sharply reduced (Fig. 5i, k). Thus, broad attachment of basal membranes to ECM, and local attachment to an equatorial contractile ring via ERM, act together to promote the efficient internalization and translocation of basal membranes driven by basal contractility (Fig. 5n). Overall, our results highlight a central role for contractility in redistributing molecules and membranes for lumen formation.

## Discussion

How embryonic cells orchestrate rapid internal remodeling required to form complex tissues and organs remains poorly understood. Here we identified two spatially and temporally distinct roles for actomyosin contractility in driving the rapid redistribution of cellular material that underlies *de novo* lumen initiation and growth. First, actomyosin contractility concentrates apical PARs at the centers of lateral cell contacts to specify the site of lumen initiation, analogous to its role in polarizing PAR proteins in other systems (36, 37). Second, we identified a novel and surprising role for basal contractility in driving the large-scale transport of basal membranes and associated material across the cytoplasm toward the apical lumen surface to enable rapid lumen growth.

The basal contractile ring in notochord cells shares many structural features with the cytokinetic contractile ring (21), and as we show here, shares key regulators, including PLK1 (28), ERM (38) and 14–3-3 (39, 40) proteins. However, as our 3D live imaging reveals, the basal contractile ring has strikingly different dynamics, which allow it to play fundamentally different roles in notochord tubulogenesis. The cytokinetic ring couples tightly and continuously to the equatorial membrane to drive furrow ingression. Previous studies suggested that the cytokinetic ring has a latent capacity for local self-repair that could make cytokinesis more robust (29). Here we show that notochord cells harness this capacity in the basal contractile ring to execute a completely novel morphogenetic function—using repeated cycles of local rupture, detachment and self-repair (Fig. 4b–d, Supplementary Movie 5), to drive the internalization and vectorial transport of basal membranes toward the apical lumen surface (Fig. 4e, Supplementary Movie 6), while maintaining itself as a force-generating structure to drive AP cell elongation (21).

Our findings also reveal new insights into how basally enriched ERM protein and ECM synergize to support apical lumen growth: equatorially enriched ERM recruits 14–3-3εa (27) to promote the high levels of contractility (Fig. 2c, 3a, Supplementary Fig. 7a) that drive local rupture/detachment and inward contraction of the basal contractile ring, and it acts as linker to couple inward contraction to membrane internalization and transport (Fig. 5b, e). While previous studies suggested that ECM signals through integrins to inhibit actomyosin contractility and promote lumen formation (1, 9, 41), our results reveal a mechanical role for ECM in anchoring the basal surface against inward forces produced by the basal contractile ring. Absent this anchor, the ring drives abnormally broad and deep invaginations of the basal surface (Fig. 5i, Supplementary Fig. 8c, Supplementary Movie 15); ring detachment is delayed, but when it does occur, the ring detaches completely from the basal surface, without internalizing basal membranes (Fig. 5i, l, Supplementary Movie 15). Thus, we propose a model in which ECM anchors the basal surface, while ERM promotes equatorial contractility and cortex/membrane coupling, to concentrate the forces that pull against this anchor to drive local mechanical rupture/detachment of the basal contractile ring and the internalization of basal membranes (Fig. 5n).

How does local rupture/detachment of the basal contractile ring drive the internalization of basal membranes? One possibility is that inward forces produced by the basal contractile ring, could act directly on the plasma membrane to induce local tearing and internalization, followed by local membrane self-repair (42). However, key regulators of endocytosis, including caveolin, dynamin, and clathrin, are essential for lumen growth in ascidians (43–45). Therefore, we favor a model in which locally enhanced endocytosis pre-internalizes a subset of basal membranes, which then couples through ERM to the internalizing contractile ring. Basal contractility could enhance local endocytosis, either by clustering endocytic machinery in the cortical plane (46–49), or in principle, by exerting forces orthogonal to the basal surface to promote scission of endocytic intermediates (50). Whether internalized membranes fuse directly with the apical surface or are first sorted and secreted via exocytic pathways (44) remains unclear. We previously observed that rapid lumen expansion sometimes coincides with pulses of basal-apical cytoplasmic flow (27), that reflect the episodic contractions described here, suggesting that actomyosin may also coordinate the fusion of newly delivered membrane to the apical domain.

Regardless of the exact mechanisms involved in the extraction and delivery of basal membranes, our work reveals a previously undescribed mode of transcytosis, driven by actomyosin contractility, that enables large-scale membrane redistribution to support rapid lumen growth. Given the broad conservation of actomyosin contractility and its regulation, we suggest that similar modes of actomyosin-based transport drive rapid morphogenetic remodeling in many other biological systems.

## Methods

### Biological materials and drug treatments.

*Ciona robusta* were collected and shipped from Half Moon Bay, Oyster Point, and San Diego (M-REP, CA). Adults were maintained under constant light to induce oocyte maturation, in oxygenated seawater at ~16°C. We used standard methods for embryo fertilization as previously described (27, 51–53). We cultured embryos at 18°C in plastic petri dishes coated with 0.1% gelatin and filled with HEPES-buffered artificial seawater (ASWH). Constructs for notochord-specific expression were electroporated into the zygote using a Gene Pulser XCell system (Biorad) or a NEPA21 (Nepa Gene). For the former, we set the parameters with 50 V, 1000 μF, infinite resistance, and 4 mm cuvette length. For the latter, we used two types of pulses: poring pulse with 70 V, 10 ms pulse length, 50 ms pulse interval, 2 pulses, 10% decay rate, and plus polarity; transfer pulse with 8 V, 50 ms pulse length, 50 ms pulse interval, 5 pulses, 40% decay rate with plus and minus polarity. For halo tag staining, JF549 (Promega) or JF669 (Promega) was added to the ASWH containing embryos at fertilization, and washed several times before the live imaging. For all drug treatments, we added drugs from stock solutions to ASWH to achieve final concentrations: 100 μM para-aminoblebbistatin (pAB) (Cayman), 10 μM UTKO1 (26, 54, 55), 10 μM H1152 (Millipore), 100 μM BI 2536 (MedChem Express) and 3 mg/mL Collagenase and Dispase (Coll+Disp) (Roche). We prepared stock solutions for pAB, UTKO1, H1152 and BI 2536 in DMSO. We held the final concentration of DMSO below 1:500 to avoid non-specific side effects, and treated control embryos with the same concentration of DMSO in ASWH. For Coll+Disp treatments, we dissolved the compounds directly into ASWH and used untreated embryos as controls. Although the concentration of BI 2536, 100 μM, is ~100-fold higher than that used for cultured cells, this was the minimum concentration at which we observed cell division defects, and we observed no developmental defects at lower concentrations (Supplementary Fig. 3a). We microinjected control and ERM morpholinos into fertilized eggs as previously described (27).

### F-actin and antibody staining.

For F-actin staining, we fixed and stained embryos with Alexa Fluor 488 Phalloidin (Thermo Fisher) as described previously (27, 52, 56). For aPKC immunostaining, we used aPKC (sc-216) antibody (Santa Cruz, CA) as described (27). For mono-phosphomyosin (1P-myosin) immunostaining, we used primary antibodies to Ser19 phospho-myosin (1:200, Cell Signaling), and followed a previous protocol (57) with one minor modification; we fixed the embryos in 2% formaldehyde, 0.1% glutaraldehyde, and 10% Trichloroacetic acid on ice. These embryos stained with F-actin or antibody were mounted in Murray clear (2:1 benzyl benzoate/benzyl alcohol), and then imaged on a Zeiss LSM 880 confocal microscope with a 40X/1.4NA (numerical aperture) oil-immersion objective.

### Constructs for notochord-specific expression of FP fusions.

We used the Gateway system to produce vectors for notochord-specific expression of various fluorescent tags. To create Gateway destination vectors for notochord-specific expression, we PCR amplified mNeonGreen, mScarlet-I, Halo tag, and mStayGold (58) and inserted them into the 5’ or 3’ entry site of a standard Gateway pFOG::RfA cassette (59) using StuI or EcoRV restriction sites. Then we inserted the notochord-specific promoter Brachyury at the HindIII site, which is located before the basal FOG (Friend of GATA) promoter. We created Gateway entry clones containing Lifeact (60), MRLC (21, 23), and a plasma membrane marker (GAP43) (59) by PCR amplification and insertion into the pCR8/GW/TOPO (Thermo Fisher) vectors using the EcoRI restriction site. We performed all insertions of PCR products into destination vectors or entry clones using the HiFi DNA assembly kits (New England BioLab). Finally, we used the Gateway LR reaction (Thermo Fisher) to recombine different entry clones into the notochord-specific destination vectors.

### 4D live time-lapse imaging.

We performed 4D live time-lapse imaging using one of the following three microscopes: A Nikon SoRa microscope equipped with a CSU-W1 scanhead and a 100x/1.46NA oil-immersion objective; A Leica SP8 confocal microscope with a 86x/1.2NA water immersion objective; and Nikon ECLIPSE-Ti inverted microscope equipped with Yokogawa CSU-X1 spinning disk and Andor iXon3 897 EMCCD camera, using a 60x/1.2 NA water immersion objective. We immobilized embryos by embedding them in 2% TopVision low melting point agarose gel (Thermo Scientific) or by anesthetizing them with 0.025% MS-222 (Sigma). We observed dynamic inward movements of actomyosin using both methods (Gel: Fig. 4a; MS-222: Supplementary Fig. 5b). We also anesthetized embryos using MS-222 to compare the speed and frequency of actomyosin movements, intensity of internalized membrane, and outward displacement of basal membrane under different chemical or genetic perturbations (Fig. 4h, i, Fig. 5c–f, 5j–m, and Supplementary Fig. 6c, d). We acquired Z stacks using x-y motorized stages and fast piezoelectric Z-axis stepper motors with 0.2–1 μm focus steps. We varied exposure times, number of Z sections, and time intervals between focus sweeps based on microscope platform and intensities of expressed probes.

### Deconvolution and visualization.

We performed all image processing and measurements using Fiji. We used deconvolution to enable visualization of detailed structures, but performed all measurements of signal intensity on raw data (see “Quantification of intensities” below). We deconvolved the images using the plugin DeconvolutionLab2 (61) or standalone software Deconwolf (62) with the point spread functions (PSFs) calculated by PSF Generator, a Fiji plugin, or measured from fluorescent beads injected in *Ciona* notochord. We used the Fiji plugin TurboReg with a custom-built macro to correct for drift in embryo positions during imaging. To visualize myosin II dynamics on the basal cortex, we constructed maximum intensity projections (MIPs) using the 24 slices closest to the basal surface. Importantly, the cytoplasmic myosin II signals were much dimmer than those coming from the basal surface, and thus they made minimal contributions to the MIPs.

### Morphological measurements.

To measure AP cell lengths, we measured the distance between lateral contacts along the basal surface. To measure lumen surface area and volume, we manually traced lumen boundaries to create ROIs for a subset of Z sections. Then, we used interpolation to estimate ROIs for all other Z positions. To quantify outward membrane displacement following ring detachment, we measured the change in basal membrane positions perpendicular to the basal surface before (t = 0) and after (t = 1 min) ring detachment from individual kymographs.

### Quantification of intensities.

We performed all measurements of signal intensity using raw data. To measure mono-phospho-myosin intensities on basal and lateral surfaces, for each cell, we determined the Z-position at which the cross-sectional lengths of lateral contacts was maximal. For basal surfaces, we manually selected ROIs (with fixed widths across all measurements), centered at the equator and encompassing the equatorial region of high intensity. For lateral contacts, we selected ROIs spanning the entire contact. For each ROI, to correct for variations in antibody staining, we measured the average pixel intensity, and divided it by the background pixel intensity measured in adjacent regions of cytoplasm from the same cells. We then scaled all measurements by the mean control value to emphasize the fold change produced by each perturbation. To measure the spatial profiles of PAR protein intensities on lateral contacts, we selected the middle section of each z-stack and measured the pixel intensity profile I(x) within an ROI spanning the contact. To correct for variations in antibody staining or FP expression levels, we normalized the raw intensity profiles I(x) measured along each contact using I(x) → (I(x) - I_min_)/I_avg_, where I_min_ and I_avg_ are the minimum intensity and the average intensity, respectively, measured along the contact. Finally, we normalized position along each contact length to facilitate alignment of data from multiple contacts. To quantify the amount of basal membrane internalized following local ring detachment, we used the F-actin signal to manually trace sections of internalized rings from 0.5–1 min after their detachment from the basal surface. Then we used those traces to measure the average intensity of the actin-colocalized membrane signal (I_col_). To correct for differences in the expression levels of membrane markers and background signals across individual cells, we measured the average signal intensity of membrane at the basal surface (I_sur_), and the average background signal outside the cells (I_bkd_). We then calculated the intensity of actin-colocalized membrane signal as (I_col_ - I_bkd_ )/(I_sur_ - I_bkd_ ). Finally, we scaled all measurements by the mean control value to emphasize the fold change produced by each perturbation.

### Quantification of dynamics.

To measure the detachment frequency and translocation speed of actomyosin, we constructed kymographs from ROIs aligned with the basal cortex as indicated in the figure schematics, and then measured the slope and timing of actin movements from the diagonal streaks of fluorescent signals.

### Statistics and reproducibility.

Statistical analyses were performed in Python. Sample size, mean, SEM, median, and first (Q1) and third (Q3) quartile values, together with the statistical tests applied, are summarized in Supplementary Table 1. Unless otherwise indicated, sample size refers to the number of cells; for Supplementary Fig. 3a, it refers to technical replicates (three independent fields of view). Statistical tests were chosen for each type of measurement (e.g., lumen volume, anterior–posterior cell length, 1P-myosin) based on whether the measurements were normally distributed, as assessed by the Shapiro-Wilk test. For normally distributed measurements, we used two-sided Welch’s t-tests to obtain t values, degrees of freedom (df), and p values, and displayed the data as dot plots with mean and SEM values. For measurements that were not normally distributed, we used two-sided Mann–Whitney U tests to obtain p values, and displayed the data as box plots with median, mean, Q1 and Q3 values, and 95% confidence intervals. For multiple comparisons, p values were adjusted using the Bonferroni correction. All experiments were independently repeated at least twice to confirm reproducibility.

## Supplementary Material

Supplementary Files

This is a list of supplementary files associated with this preprint. Click to download.

• SupplementaryTable1.xlsx

• MizotaniandMunroSupplNatCommun2025.pdf

• MovieS1.mp4

• MovieS2.mp4

• MovieS3.mp4

• MovieS4.mp4

• MovieS5.mp4

• MovieS6.mp4

• MovieS7.mp4

• MovieS8.mp4

• MovieS9.mp4

• MovieS10.mp4

• MovieS11.mp4

• MovieS12.mp4

• MovieS13.mp4

• MovieS14.mp4

• MovieS15.mp4

• MovieS16.mp4

• MovieS17.mp4

## Data Availability

All data supporting the findings of this study are included in the main text, including the main figures and Supplementary Information. Raw image files are available from the authors upon request.
